# Successful Atrial Septal Defect Closure Subsequent to Medical Pulmonary Preconditioning in an Infant With Severe Pulmonary Hypertension Associated With Bronchopulmonary Dysplasia

**DOI:** 10.7759/cureus.57290

**Published:** 2024-03-30

**Authors:** Maki Sato, Hirofumi Saiki, Kanchi Saito, Akira Sato, Seiko Kuwata, Satoshi Nakano, Junichi Koizumi, Kotaro Oyama, Manami Akasaka

**Affiliations:** 1 Neonatology and Pediatrics, Iwate Medical University, Shiwa, JPN; 2 Pediatric Cardiology, Iwate Medical University, Shiwa, JPN; 3 Cardiovascular Surgery, Iwate Medical University, Shiwa, JPN; 4 Pediatrics, Michinoku Medical Center on Disability and Health, Shiwa, JPN; 5 Pediatrics, Iwate Medical University, Shiwa, JPN

**Keywords:** pediatric pulmonary hypertension, premature chronic lung disease, atrial septal defect (asd), bronchopulmonary dysplasia, extremely low birth weight

## Abstract

While atrial septal defect (ASD) may contribute to right ventricular decompression in patients with severe pulmonary hypertension (PH), the pulmonary vasculature might be compromised by increased pulmonary blood flow, even though pulmonary vasodilators successfully reduce resistance. ASD closure is a treatment option that may ameliorate PH symptoms associated with bronchopulmonary dysplasia (BPD) in infants. However, the feasibility of ASD closure is obscure in patients with BPD-PH causing right-to-left shunting.

Here, we present an eight-month-old girl with ASD complicated by BPD-PH, in which the pulmonary pressure exceeded the systemic pressure; the ASD was successfully closed after pulmonary preconditioning with dexamethasone and high-dose diuretics. Our patient was delivered as the third baby in triplets at a gestational age of 25 weeks, with a birth weight of 344 g. She was diagnosed with BPD at three months of age (37 weeks of postmenstrual age) with a body weight of 1.4 kg. Mild pulmonary hypertension was identified at the age of five months, and oral sildenafil was initiated. While her atrial septal defect was small at the time of PH diagnosis, it became hemodynamically significant when she grew up to 3.4 kg of body weight, at seven months after birth. Her estimated right ventricular pressure was apparently more than the systemic pressure, and oxygen saturation fluctuated between 82% and 97% under oxygen supplementation due to bidirectional interatrial shunt with predominant right-to-left shunting. Pulmonary preconditioning lowered the estimated right ventricular pressure to almost equal the systemic pressure and elevated arterial oxygen saturation while also suppressing right-to-left shunting. Cardiac catheterization after preconditioning revealed a ratio of pulmonary blood pressure to systemic blood pressure ratio (Pp/Ps) of 0.9, pulmonary resistance of 7.3 WU-m^2^, and a pulmonary to systemic blood flow ratio (Qp/Qs) of 1.3 (approximately 1.0 in the normal circulation without significant shunt), with the cardiac index of 2.8 L/min/m^2^. The acute pulmonary vasoreactivity test against the combination of 20 ppm nitric oxide and 100% oxygen was negative, although the patient had consistently high pulmonary flow with makeshift improvements after preconditioning. Despite the high pulmonary resistance even after preconditioning, aggressive ASD closure was performed so that pulmonary flow could be consistently suppressed regardless of the pulmonary condition. Her Pp/Ps under 100% oxygen with 20 ppm nitric oxide was 0.7 immediately after closure. After two years of follow-up, her estimated right ventricular pressure was less than half of the systemic pressure with the use of three pulmonary vasodilators, including sildenafil, macitentan, and beraprost. A strategy to temporarily improve PH and respiratory status aimed at ASD closure could be a treatment option for the effective use of multiple pulmonary vasodilators, by which intensive treatment of BPD can be achieved.

## Introduction

Bronchopulmonary dysplasia (BPD) is a chronic lung disease in infants, characterized by impaired angiogenesis and alveolarization, resulting in abnormal pulmonary vascular function because of chorioamnionitis, prenatal infection, impaired lung developmental modulation, and postnatal lung injury [1]. Approximately half of the infants delivered between 22 and 29 weeks of gestational age develop this condition, and its prevalence and severity are associated with decreasing gestational age at birth, as well as respiratory support requirements during neonatal care [2]. Although the range of pulmonary hypertension (PH) and respiratory compromise is broad because the current diagnostic criteria include any form of respiratory support requirement at 36 weeks of postmenstrual age, PH derived from bronchopulmonary dysplasia (BPD) is associated with high mortality, high frequency of hospital admission, and impaired neurodevelopmental outcomes [3]. A pronounced incidence of BPD has been reported in premature infants with atrial septal defects (ASD) [4]. ASD closure may ameliorate BPD symptoms in both acute and chronic situation [5-7], suggesting a beneficial impact of suppressing pulmonary blood flow and its fluctuation. Despite the beneficial impact of closing the ASD, there are cases in which the general condition is extremely compromised, and PH is too severe to close it. We report a case of severe BPD-associated PH (BPD-PH) complicated by ASD, in which both suppression of right ventricular (RV) pressure and improved oxygenation, partly because of suppressed right-to-left shunting, were temporarily observed due to a combination of dexamethasone and high-dose diuretics in the perioperative period, and surgical ASD closure was successfully performed. This case is novel since we were challenged to close an ASD in a patient with severe PH causing right-to-left shunting by using aggressive medical preconditioning. Our case highlights the importance of pursuing the possibility of ASD closure by simulating the best possible pulmonary condition using pulmonary preconditioning, which allows intensive use of pulmonary vasodilators without concern for exacerbating pulmonary high flow.

## Case presentation

An eight-month-old girl with ASD was referred to us for the management of pulmonary hypertension due to BPD. She was delivered as the third baby in a triplet at a gestational age of 25 weeks. The patient weighed 344 g at birth. Owing to respiratory distress syndrome, she was intubated, and pulmonary surfactant was administered. Two days after birth, the ductus arteriosus was closed after indomethacin administration. She was extubated at two months old, after which she required 30-40% oxygen to maintain a blood oxygen saturation of more than 93%. Mild pulmonary hypertension was identified at the age of five months, with a body weight of 2.1 kg. Because the ASD was as small as 3.8 mm, oral sildenafil was introduced at this point. She was discharged from the hospital at seven months of age, which corresponds to a corrected postnatal age of three months. She was on budesonide inhalation and home-based oxygen therapy.

**Figure 1:**
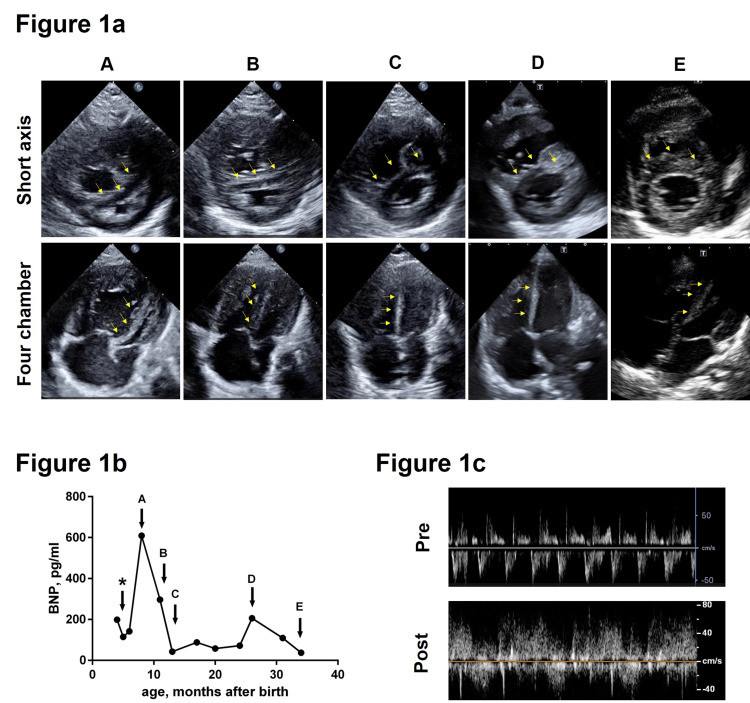
Patient's clinical course Figure 1a: Chronological changes in the shape of interventricular septum during systole Chronological changes in the interventricular septum shape (yellow arrow) during systole are shown. A: at the first clinical visit to Pediatric Cardiology, B: after pulmonary preconditioning, C: after atrial septal defect closure, D: at the time of respiratory infection requiring intubation, E: 2 years after the operation. Note that the interventricular septum during systole deviated to the left ventricle before preconditioning whereas it inverted to deviated to the right ventricle after atrial septal defect repair. Figure 1b: The trend in plasma B-type natriuretic peptide levels is shown. A-E corresponds to A-E in Figure 1a. Asterisk indicates the initiation of sildenafil in the neonatal intensive care unit (NICU). Figure 1c: Blood flow patterns of the atrial septal defect before and after pulmonary preconditioning are shown. The flow pattern of the interatrial communication was consistently bidirectional throughout the clinical course. However, right-to-left shunting was predominant at the first clinical visit (upper panel, which corresponds to A in Figures 1a-1b), whereas it became left-to-right shunting predominant after preconditioning (lower panel, which corresponds to B in Figures 1a-1b).

**Figure 2:**
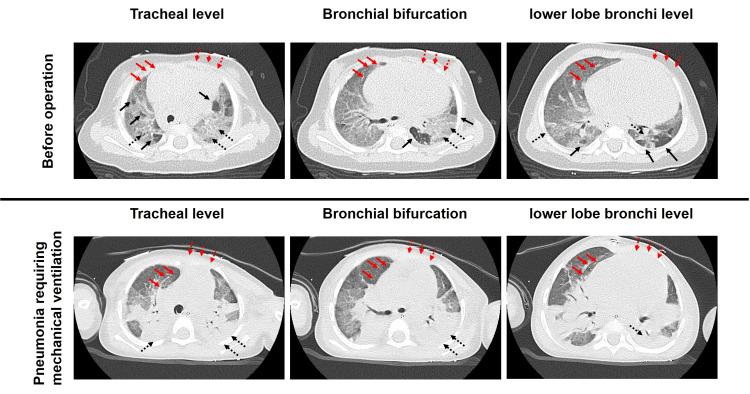
Chest computed tomography images before pulmonary preconditioning and at the time of respiratory infection Upper panel: Chest computed tomography images before pulmonary preconditioning (upper panel, corresponding to A in Figures 1a-1b) are shown. A mixture of emphysema (black solid arrows) and compromised aeration/atelectasis (black dotted arrows) is observed in the lung field. An enlarged shape of the right atrium (red solid arrow) and right ventricle (red dotted arrow) are observed. Lower panel: Chest computed tomography images during respiratory infection (lower panel, corresponding to D in Figure 1a-1b) are shown. Diffused atelectasis is observed (black dotted arrow); however, the right ventricular systolic pressure is not as high as before (D in Figure 1a). Note that the enlargement of the right atrium (red solid arrow) and right ventricle (red dotted arrow) are markedly attenuated compared with the images in the upper panel.

At her first clinic visit to the pediatric cardiology department, she was eight months old with a body weight of 3.4 kg, and arterial oxygen saturation (SatO2) fluctuated between 82% and 97% with nasal oxygen supplementation due to both BPD and bidirectional shunt of the ASD (Figures 1a-1b). Her respiratory rate was approximately 60 breaths per minute and mild retraction was observed. Her ASD was 7.3 mm×12.4 mm in diameter. The transtricuspid regurgitation pressure gradient (TRPG) was consistently > 65 mmHg with marked inferior vena cava dilatation, suggesting severe PH. Systemic systolic blood pressure was approximately 65 mmHg, and marked intraventricular compression by the right ventricle (RV) suggested oversystemic PH (A in Figure 1a). The RV diastolic diameter was 204% of normal body size. The left ventricular (LV) end-diastolic diameter was only 65% of its normal size due to LV compression caused by RV volume and pressure loads (Figure 1a). Chest computed tomography revealed BPD, with emphysema and pulmonary consolidation (Figure 2, upper panel).

The patient failed to thrive. She experienced frequent vomiting. Plasma BNP levels increased from 142 pg/ml to 609 pg/ml, and PH was exacerbated by the use of sildenafil (A in Figures 1a-1b). Therefore, the sildenafil treatment was discontinued at the initial clinical visit (A in Figures 1a-1b). In two months (six months of corrected postnatal age), active nutrition via the nasogastric tube became possible, and her body weight increased to 4.2 kg, but her peak SaO2 decreased from 97% to 92% under the same oxygen supplementation, and TRPG increased to 80 mmHg. We sought the possibility of ASD closure aimed at preventing BPD progression and stabilizing SatO2 at the expense of losing the potential source of RV decompression [2-4].

Since her RV pressure was apparently more than systemic pressure (A in Figure 1a), we prescribed intravenous dexamethasone with 0.3 mg/kg/day for five days in order to observe the possibility of temporal improvement of lung condition. This dose was determined by traditional dosing with dexamethasone for the treatment of BPD [8]. She was also administered high-dose diuretics (furosemide/spironolactone 2 mg/kg/day, tolvaptan 0.4 mg/kg/day) to improve the left ventricular diastolic function via right ventricular decompression [9]. Her peak SatO2 improved from 92% to 95% under the same oxygen supplementation and the shape of the interventricular septum suggested slightly lowered pulmonary pressure (B in Figure 1a). The component of right-to-left shunting via ASD decreased, and the left-to-right component became predominant (Figure 1c) after preconditioning. Cardiac catheterization was performed to evaluate pulmonary circulation and vasoreactivity [10]. The pulmonary resistance with 25% oxygen was 7.3 WU-m^2^, with Pp/Ps of 0.9, which was lowered to 6.3 WU-m^2^ and 0.7, respectively, both with 100% oxygen and 20ppm of nitric oxide (NO) inhalation. The ratio of pulmonary blood flow to the systemic blood flow (Qp/Qs) and mean pulmonary pressure changed from 1.35 and mean 35 mmHg, to 1.28 and 29 mmHg, respectively. The cardiac index was 2.8 L/min/m^2^ before and after the vasoreactivity test. These results confirm the existence of consistently high pulmonary blood flow under transient improvement of the pulmonary condition by preconditioning, although high pulmonary resistance is not favorable for ASD closure [10]. Based on these findings, we decided to proceed with surgical ASD closure to prevent further insults from the high pulmonary blood flow.

The ASD was an inferior rim defect with a size of 15 ×10 mm; thus, it was surgically closed using an auto-pericardial patch. The mean pulmonary pressure immediately after ASD closure was 35 mmHg with a Pp/Ps of 0.7 under 100% oxygen and NO inhalation. NO inhalation was converted to sildenafil, and the patient was extubated on postoperative day (POD) 2. Echocardiography revealed a reduced pulmonary pressure (C in Figure 1a). The patient was discharged from the hospital on POD 15. Her SatO2 was 100% under oxygen supplementation (0.5 L/min), and macitentan was prescribed before discharge. She was then administered sildenafil (2 mg/kg/day), macitentan (0.4 mg/kg/day), and beraprost (2μg/kg/day) as outpatients. Even under respiratory infection requiring mechanical ventilation (Figure 2, lower panel), her estimated RV pressure was less than the systemic pressure (D Figure 1a), although the plasma BNP level mildly increased (D in Figure 1b). Two years after ASD closure, the estimated pulmonary artery pressure was at most half of the systemic pressure (E in Figure 1a), and SatO2 was 95-98% even without oxygen supplementation, although home oxygen therapy was continued to minimize reactive pulmonary hypertension.

## Discussion

In cases of severe pulmonary hypertension, persistent interatrial communication exerts right ventricular decompression via a right-to-left shunt, which is attributed to the preservation of cardiac output. Therefore, closure is not recommended if a right-to-left shunt is present in adults. Conversely, particularly in children, the treatment of PH in the presence of ASD may result in increased pulmonary blood flow and accelerate the progression of pulmonary vascular disease. Since the estimated survival of BPD-PH in 2 years is reported to be 53% [11], we decided to aggressively close the ASD to maximize the possibility of preventing BPD worsening.

Acute and chronic benefits of ASD closure in patients with BPD have been reported [5-7]. In particular, Thomas et al. reported marked amelioration of the general condition in 11 of 13 patients after ASD device closure and some of them exhibited suppressed RV pressure and reduced oxygen dependency [6]. Webb et al. observed clinical symptoms before and after ASD closure using the BPD-PH score and found that ASD closure in patients with BPD-PH reduces diuretic use and respiratory support requirements [7]. Although a high procedural success rate of transcatheter closure was reported in a cohort with an average body weight of 8 kg and less than 24 months of age, approximately 10% of the patients died [6,12], suggesting the need for meticulous management after ASD closure, even without cardiopulmonary bypass. Compared with studies that challenged ASD closure in patients with severe BPD-PH [5-7], the birth weight in our case was minimal, and prematurity estimated by gestational age at delivery was relatively young, suggesting that this case is among the most severe cases reported. Importantly, in contrast to other reports, our patient was a candidate for surgical ASD closure requiring cardiopulmonary bypass due to an inferior rim defect. Accordingly, we performed preconditioning before hemodynamic assessment with postoperative maximum pulmonary vasodilators.

In anticipating the potential of improving BPD with adequate nutrition and growth, we assumed that ASD closure was not only possible but also preferable based on the catheter data, regardless of high pulmonary resistance exceeding the recommended pulmonary resistance of less than 6 WU-m^2^ according to the guideline [10]. Indeed, while all the BPD-PH patients experienced ASD closure in the prospective cohort published by Webb et al. and fulfilled guidelines for closing ASD, the authors challenged the possibility of closing ASD in the patients with BPD-PH in the discussion section, considering the disease-specific properties of lung and heart [7]. Due to inferior rim deficiency, our patient underwent surgical closure, but her perioperative course was uneventful under pulmonary preconditioning with preoperative dexamethasone and postoperative pulmonary vasodilators. 

In patients with BPD-PH, sildenafil is among the recommended pulmonary vasodilators [3]. Before ASD closure, we hesitated to continue sildenafil because of vomiting, a known complication, as well as concern for increasing pulmonary blood flow. The worsening of RV enlargement before referral convinced us of the possibility of an untoward effect of sildenafil in combination with ASD and BPD-PH (A in Figures 1a-1b). While the RV pressure decreased to approximately 70% of the systemic pressure after ASD closure, we further added macitentan and beraprost because the patient no longer had atrial communication. Despite efforts to avoid infection, she developed a respiratory infection requiring mechanical ventilation, but her estimated RV pressure never increased to supersystemic pressure (D in Figure 1a).

The use of steroids remains controversial. Considering severe fetal growth retardation with prematurity at 25 weeks of gestational age, our patient had factors suggestive of lung developmental arrest, i.e. so-called “New BPD” [13], which is supposed to be less responsive to steroids. Meanwhile, the requirement for oxygen more than 10 months after birth coupled with malnutrition implied factors compatible with so-called “old BPD”. Although the histological assessment was unavailable due to the patient's severely compromised general condition, the possibility of having both characteristics of “New” and “Old” BPD allowed us to try preconditioning involving dexamethasone. Dexamethasone has been suggested as an effective treatment for acute exacerbation of chronic lung disease [8]. We also took advantage of using dexamethasone simultaneously as lung preconditioning for subsequent surgical ASD closure. Although dexamethasone use for infants undergoing cardiac surgery did not reduce mortality or major complications compared with placebo in the DECISION Randomized Clinical Trial [14], patients with PH are at an increased risk for perioperative complications, which rationalizes the use of steroids in this patient [15]. Accordingly, immediately after verifying the effectiveness of pulmonary preconditioning on PH suppression, she was referred for surgical ASD closure, taking advantage of preoperative dexamethasone loading.

The optimal timing of specialized care by pediatric cardiologists is difficult. There are concerns regarding the safety of cardiopulmonary bypass in preterm and low-birth-weight infants, although it is well-known that many ASDs are asymptomatic. The benefits and drawbacks of pulmonary vasodilators in the presence of ASD are unknown, particularly in infants, where the right ventricle is still relatively stiff. In principle, interventions should be considered when the PH begins to progress. Therefore, it is important to accumulate more cases with meticulous hemodynamic assessments.

## Conclusions

In patients with BPD-PH, pulmonary preconditioning with dexamethasone and high-dose diuretics was effective in temporarily suppressing PH, improving oxygenation, and reducing right-to-left shunting, which allowed for uneventful surgical ASD closure in combination with postoperative pulmonary vasodilators. While preconditioning should be considered for severe cases, it is also important to consider treatment before the disease becomes severe. Although this preconditioning was effective in this case, other modes of tailor-made preconditioning may be necessary in other cases and may need to be considered depending on the pathological condition. As ASD closure rationalizes aggressive pulmonary vasodilatation therapy, efforts to seek the possibility of closing the ASD are vitally important in ASD patients with BPD-PH.
